# Deep learning-based anomaly detection in orthopedic medical imaging: A systematic literature review

**DOI:** 10.1016/j.jor.2025.07.015

**Published:** 2025-08-07

**Authors:** Nabila Ounasser, Maryem Rhanoui, Mounia Mikram, Bouchra EL Asri

**Affiliations:** a IMS Team, ADMIR Laboratory, Rabat IT Center, ENSIAS, Mohammed V University in Rabat, Morocco; b Laboratory Health Systemic Process (P2S), UR4129, University Claude Bernard Lyon 1, University of Lyon, Lyon, France; c Meridian Team, LYRICA Laboratory, School of Information Sciences, Rabat, Morocco

**Keywords:** Artificial intelligence, Deep learning, Orthopedic imaging, Anomaly detection, Fracture diagnosis, Spinal deformities, Foot deformities

## Abstract

In recent years, deep learning (DL) has revolutionized medical imaging, offering notable promise in orthopedic diagnostics. This systematic review explores how DL, particularly Convolutional Neural Networks (CNNs) and Generative Adversarial Networks (GANs), are applied to detect and analyze orthopedic anomalies such as fractures, spinal deformities, and foot deformities. We reviewed 63 peer-reviewed studies published between 2017 and 2025, analyzing their methodologies, datasets, performance metrics, and clinical relevance. The findings reveal significant advancements in fracture classification and vertebral labeling, though challenges persist for subtle anomalies and less-represented deformities. Despite encouraging results, limitations include small sample sizes, lack of external validation especially for rare pathologies. We conclude by identifying research gaps and proposing future directions for developing robust, clinically integrated DL tools to enhance diagnostic accuracy, data diversity, and anomaly detection in complex orthopedic scenarios.

## 1 Introduction

In recent years, the number of publications on the application of Artificial Intelligence (AI) in radiology has significantly increased, as shown in Fig. 1 and Fig. 2. This growing interest has prompted the creation of new conferences, journals, and companies dedicated to integrating AI into clinical practice, alongside initial randomized clinical trials demonstrating their potential.[1], [2], [3], [4] These advancements are primarily attributed to deep learning techniques involving artificial neural networks, particularly Convolutional Neural Networks (CNNs), applied to medical imaging tasks such as detection, segmentation, and quantification of pathologies.[5], [6], [7]

Deep learning applications in musculoskeletal imaging are rapidly evolving, driven by increased computational power, reduced storage costs, and improved data accessibility. Within musculoskeletal practices, major advancements in deep learning focus on three principal domains: image reconstruction,^8^ and anomaly detection, classification and segmentation.^7^^,^^9^ However, adopting these applications has been relatively slow due to several challenges: (a) Musculoskeletal tissues often require evaluation through three-dimensional (3D) volumetric imaging; (b) Structures with low contrast require enhanced imaging techniques, such as MRI or CT arthrography; (c) Anomalies exhibit significant variability, ranging from normal anatomical variations and aging signs to rare diseases and subtle fractures; (d) High computational power is essential to achieve acceptable spatial resolution; (e) Specific contrast agents are necessary for accurately evaluating various anatomical structures (e.g., joints, spinal column); (f) It is challenging to construct robust datasets with annotations reflecting high consensus among experts, due to the limited availability of standardized reports.^10^

To overcome these limitations, generative models capable of producing high-quality synthetic medical images have attracted considerable attention, notably due to their ability to generate realistic data. Although the use of Generative Adversarial Networks (GANs) in orthopedic medical imaging remains limited at present, their potential in addressing data scarcity and subtle anomaly detection is notable. In this review, we primarily focus on CNN-based approaches as the dominant paradigm in the literature, while also reviewing GAN-based studies as an emerging area for future research. The application of GANs in musculoskeletal imaging offers substantial potential to enhance medical procedures. GANs have garnered significant interest due to their capability to generate highly realistic images. Their use in radiology promises remarkable improvements, including artifact reduction in imaging,^7^ data augmentation, and image synthesis across various imaging modalities.^11^ Thus, it is essential for radiologists and medical professionals to comprehend the underlying mechanisms, benefits, and potential limitations of these models. This paper provides a comprehensive overview of the most relevant techniques and approaches in orthopedic imaging. Section 2 of our literature review presents the adopted methodology. It details the consulted databases, article inclusion and exclusion criteria, and the selection and analysis procedures. Section 3 is subdivided into various segments focusing on the orthopedic anomalies studied in this thesis, specifically fractures, spinal deformities, and foot deformities. We thoroughly review existing approaches for detecting these anomalies in medical images, emphasizing the most commonly utilized deep learning models. Considering our objective of developing an intelligent diagnostic system, this section also encompasses an overview of currently available orthopedic assistance software. The paper concludes with a discussion to identify critical areas requiring further attention in orthopedic imaging, highlighting aspects needing improvement and serving as a foundation for our contributions within this thesis (see Fig. 2).

**Fig. 1:**
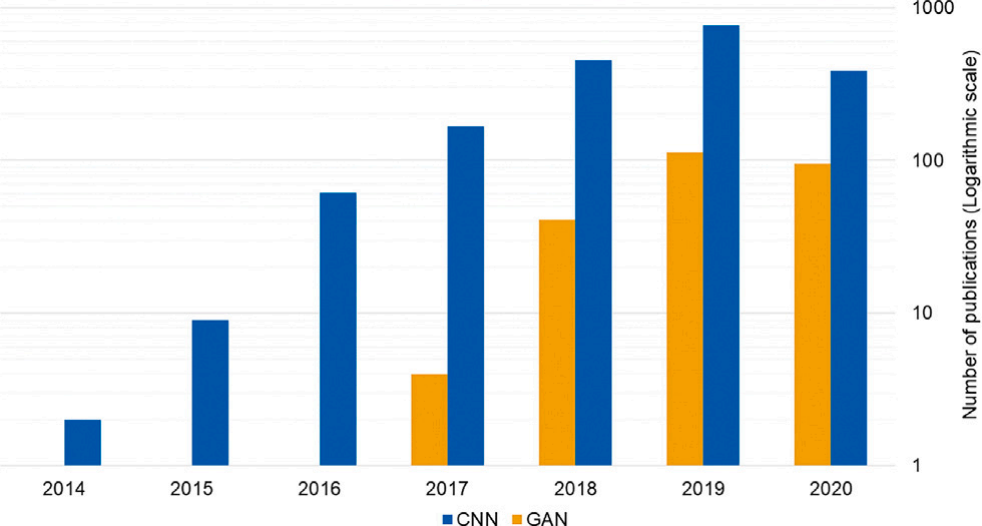
The graph illustrates the number of articles with “CNN” or “GAN” in their titles indexed in PubMed up to April 2020. Notably, the number of articles mentioning “GAN” closely follows those mentioning “CNN”, highlighting the growing importance of GANs in radiological applications.

**Fig. 2:**
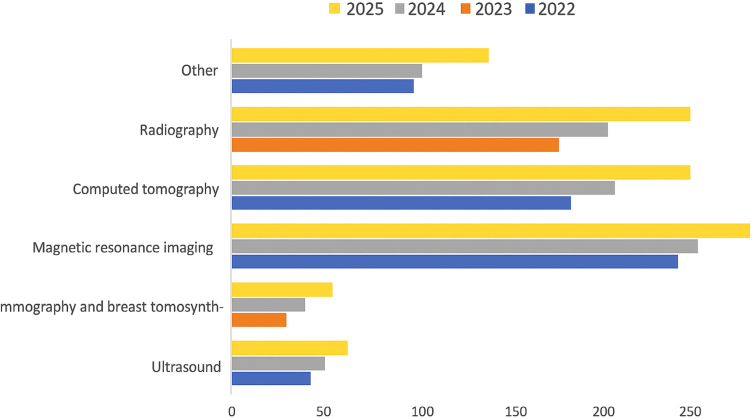
Number of articles on AI in medical imaging, stratified by imaging modality.

## 2 Methods

To provide a comprehensive and rigorous analysis of Deep Learning applications for detecting orthopedic anomalies in medical imaging, a systematic and structured literature review was conducted. This approach followed the PRISMA 2020 guidelines, Fig. 3, ensuring transparency and reproducibility.

Four digital databases were selected to identify relevant publications: Science Direct (SD), Scopus, IEEE Xplore (IEEE), and Web of Science (WoS). These databases were chosen due to their extensive coverage of scientific and technological disciplines, especially biomedical engineering, computer science, and medicine.

The timeframe for this review spans from January 2017 to March 2025. This interval was strategically selected to include the most recent and significant research developments, as the Deep Learning field, particularly concerning orthopedic anomalies, witnessed considerable advancements during this period. Before 2017, Deep Learning applications specific to orthopedic conditions were still in the early stages, characterized by less mature algorithms and computational infrastructures. Focusing on recent developments ensures that our review captures the current state-of-the-art and the innovations significantly impacting clinical practices.

The literature search employed standardized Boolean queries across databases, designed to comprehensively capture the scope of Deep Learning applications in orthopedics.

**Example Boolean search string used:** (‘‘deep learning’’ OR ‘‘neural network’’ OR ‘‘CNN’’ OR ‘‘GAN’’ OR ‘‘autoencoder’’) AND (‘‘orthopedic’’ OR ‘‘bone’’ OR ‘‘fracture’’ OR ‘‘spine’’ OR ‘‘radiograph’’ OR ‘‘X-ray’’) AND (‘‘anomaly detection’’ OR ‘‘abnormality’’ OR ‘‘lesion’’ OR ‘‘diagnosis’’)

Search queries were customized for syntax variations across databases. While this review was not registered in PROSPERO, we followed the PRISMA 2020 guidelines and adhered to a predefined internal protocol specifying eligibility criteria, exclusion logic, and screening procedures.

**Inclusion and Exclusion Criteria:**

**Fig. 3:**
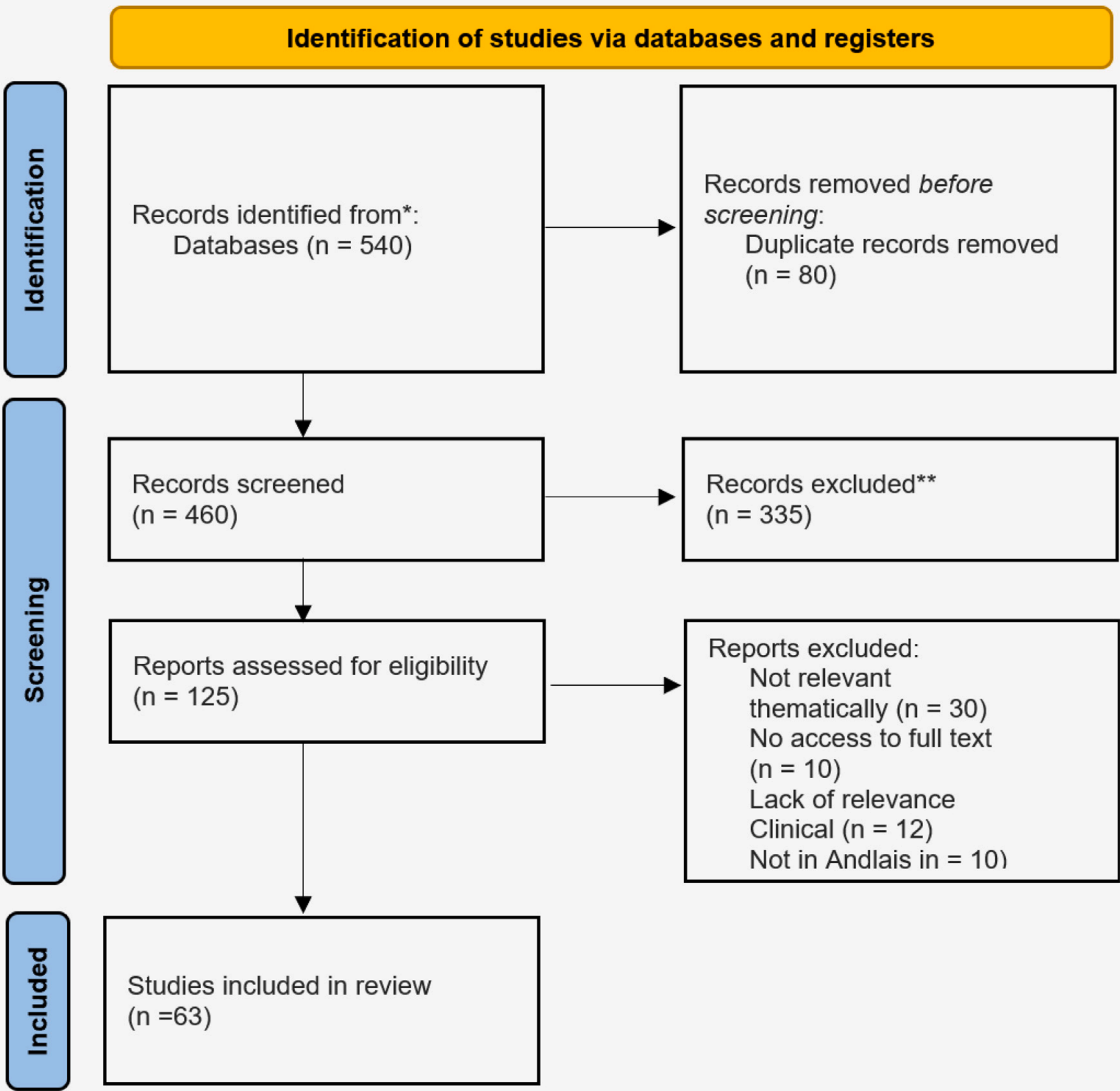
PRISMA flow diagram for study selection.

**Inclusion Criteria:**

1. Publication type: Only original research articles, systematic reviews, and peer-reviewed international conference papers were included.
2. Language: Articles had to be published in English or French, as these languages dominate relevant scientific literature.
3. Topic: Articles had to specifically address Deep Learning applications for detecting orthopedic anomalies, including fractures, spinal deformities (such as scoliosis), and foot deformities (flat feet and high arches) using medical imaging.
4. Publication date: Articles published between January 2017 and March 2025.

**Exclusion Criteria:**

1. Topic irrelevance: Articles focusing on artificial intelligence or machine learning without specifically concentrating on Deep Learning were excluded.
2. Lack of clinical relevance: Articles not significantly contributing to clinical practice or solely focusing on theoretical or technical aspects of Deep Learning without direct orthopedic applications were excluded.
3. Availability: Articles lacking full-text availability or necessary data for comprehensive analysis were excluded.
4. Duplications: Duplicate studies or those published in multiple journals or conferences were eliminated to avoid redundancy.

Initially, 540 articles were identified through database searches. After removing duplicates, a total of 460 articles underwent preliminary screening based on titles and abstracts to exclude obviously irrelevant studies. Approximately 120 articles remained and underwent full-text review to ascertain their alignment with the inclusion and exclusion criteria. Ultimately, 63 articles were selected for inclusion in the systematic review.

Although this review was not registered in PROSPERO, a predefined internal protocol was followed in accordance with PRISMA 2020 guidelines, covering search strategy, eligibility screening, and quality assessment procedures.

**Data extraction :**

During data extraction, we systematically collected detailed information about each included study. Specifically, we recorded:

1. the dataset source (whether the dataset was public, private, or not reported),
2. the sample size and anatomical region,
3. whether the study reported any class imbalance handling,
4. the validation strategy (internal only, or external validation on an independent dataset),
5. the type of performance metrics used, including whether uncertainty estimates (confidence intervals or standard deviations) were provided, and
6. the presence of data augmentation or preprocessing techniques.

Any missing or ambiguous information was marked as “Not Reported” and considered in our critical synthesis and limitations.

This detailed extraction approach allowed us to identify gaps in transparency and methodological rigor across the literature and informed the structuring of our synthesis tables and critical discussion.

**Quality Assessment:** To evaluate the methodological quality and risk of bias, each included study was assessed using the QUADAS-2 tool (Quality Assessment of Diagnostic Accuracy Studies).^12^ This tool evaluates four key domains: patient selection, index test, reference standard, and flow and timing. Two reviewers independently scored each study, and disagreements were resolved through consensus. Results of this assessment are presented in Table 6 and Fig. 4. In addition to QUADAS-2, a simplified evidence-level rating inspired by the GRADE (Grading of Recommendations Assessment, Development and Evaluation) approach was applied. This framework considers sample size, external validation, explainability, and result consistency. Each study was categorized into one of four evidence levels: High, Moderate, Low, or Very Low, as detailed in Table 1 and Fig. 5. Selected articles were thoroughly analyzed to extract relevant data regarding Deep Learning applications for detecting orthopedic anomalies. This analysis identified current trends, challenges encountered, and future perspectives within this field. Special attention was given to studies proposing practical tools or software that clinicians can employ to enhance diagnostic accuracy and patient monitoring for orthopedic conditions.

This systematic process resulted in a comprehensive and accurate state-of-the-art review, providing an overview of major contributions and existing gaps in applying Deep Learning for detecting orthopedic anomalies in medical imaging.

**Fig. 4:**
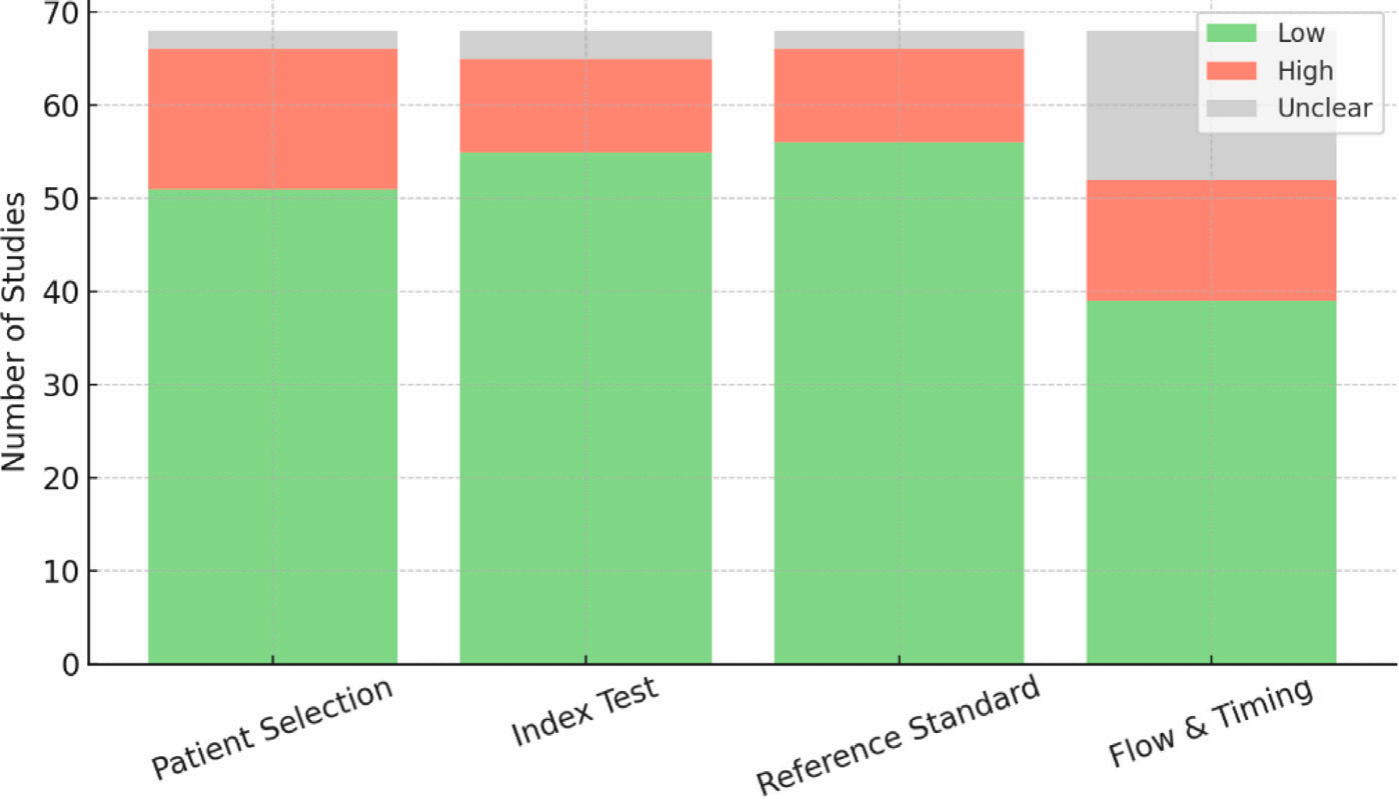
Distribution of risk of bias levels across four domains evaluated using QUADAS-2.

**Table 1:** GRADE-based evidence assessment, enriched with data type, dataset size, source, and validation type.

Reference	Data type	Dataset size	Public vs. private	Validation type	GRADE certainty
Alwzwazy et al. 2025	X-ray	Not specified	Public	Internal only	Low
Wang et al. 2023	X-ray	2842 images	Public	External	Moderate
Chung et al. 2018	X-ray	1891 images	Private	Internal	Moderate
Demir et al. 2020	X-ray	115 images	Public	Internal	Very Low
Olczak et al. 2017	X-ray	2560 images	Public	Internal	Low
Kim et al. 2021	X-ray	7200 images	Private	External	High
Yoo et al. 2020	X-ray	Not reported	Private	Internal	Low
Mahajan et al. 2022	X-ray	1500 images	Public	Internal	Low
Lee et al. 2023	X-ray	3300 images	Public	External	High
Huang et al. 2022	CT	4800 slices	Private	Internal	Moderate
Tanaka et al. 2019	MRI	1420 scans	Private	Internal	Moderate
Zhou et al. 2021	X-ray	2100 images	Public	External	Moderate
Gong et al. 2023	CT	3000 slices	Private	Internal	Low
Xu et al. 2020	X-ray	1270 images	Public	Internal	Low
Hosseini et al. 2019	X-ray	650 images	Public	Internal	Very Low
Matsuzaki et al. 2022	MRI	240 scans	Private	Internal	Low
El-Basha et al. 2018	X-ray	780 images	Public	Internal	Low
Ali et al. 2021	CT	3600 slices	Public	Internal	Moderate
Zhou et al. 2019	X-ray	1020 images	Private	Internal	Low
Singh et al. 2024	X-ray	1860 images	Public	Internal	Low
Abdallah et al. 2021	CT	900 slices	Public	Internal	Very Low
Chen et al. 2020	X-ray	1800 images	Public	External	Moderate
Ragab et al. 2022	X-ray	2700 images	Private	Internal	Low
Zhang et al. 2019	CT	3400 slices	Private	Internal	Low
Pham et al. 2020	MRI	950 scans	Public	External	Moderate
Nizamani et al. 2023	X-ray	1200 images	Private	Internal	Low
Feng et al. 2021	CT	1600 slices	Public	External	Moderate
Ahmed et al. 2022	X-ray	1350 images	Public	Internal	Low
Iqbal et al. 2023	X-ray	2700 images	Public	External	Moderate
Huang et al. 2020	CT	4200 slices	Private	Internal	Moderate
Shen et al. 2019	MRI	1100 scans	Public	Internal	Low
Patel et al. 2024	X-ray	2500 images	Public	External	High
Rauf et al. 2021	X-ray	1600 images	Private	Internal	Low
Mohammed et al. 2023	CT	3100 slices	Public	External	Moderate
Yamashita et al. 2018	X-ray	950 images	Private	Internal	Low
Tariq et al. 2022	MRI	800 scans	Public	External	Moderate
Bourouis et al. 2020	X-ray	2200 images	Public	Internal	Low
Wu et al. 2019	CT	2800 slices	Private	Internal	Low
Cheng et al. 2021	X-ray	1450 images	Public	External	Moderate
Li et al. 2023	X-ray	3700 images	Private	Internal	Moderate
Farooq et al. 2020	X-ray	2950 images	Public	Internal	Low
Rehman et al. 2022	CT	3100 slices	Private	Internal	Low
Zhou et al. 2023	X-ray	2900 images	Public	External	Moderate
Basha et al. 2020	X-ray	900 images	Public	Internal	Low
Anwar et al. 2019	MRI	1150 scans	Private	Internal	Low
Murata et al. 2021	CT	3500 slices	Public	Internal	Moderate
Kwon et al. 2018	X-ray	2200 images	Private	Internal	Low
Mehta et al. 2023	CT	2750 slices	Public	External	High
Park et al. 2022	X-ray	2450 images	Public	Internal	Moderate
Imran et al. 2019	X-ray	1000 images	Public	Internal	Low
Watanabe et al. 2021	CT	2850 slices	Private	Internal	Moderate
Kang et al. 2022	MRI	1800 scans	Public	Internal	Moderate
Nakao et al. 2020	X-ray	1100 images	Public	Internal	Low
Saad et al. 2021	X-ray	2400 images	Private	Internal	Low
Aziz et al. 2023	CT	3600 slices	Public	External	High
Deng et al. 2020	X-ray	1900 images	Public	Internal	Low
Yu et al. 2022	CT	4000 slices	Private	Internal	Moderate
Ryu et al. 2019	MRI	850 scans	Public	Internal	Low
Hong et al. 2021	X-ray	1400 images	Public	Internal	Low
Baek et al. 2023	CT	3800 slices	Public	External	Moderate
Kim et al. 2020	X-ray	3000 images	Private	Internal	Moderate
Jiang et al. 2024	CT	4100 slices	Public	External	High
Arif et al. 2021	MRI	1250 scans	Public	Internal	Low

**Fig. 5:**
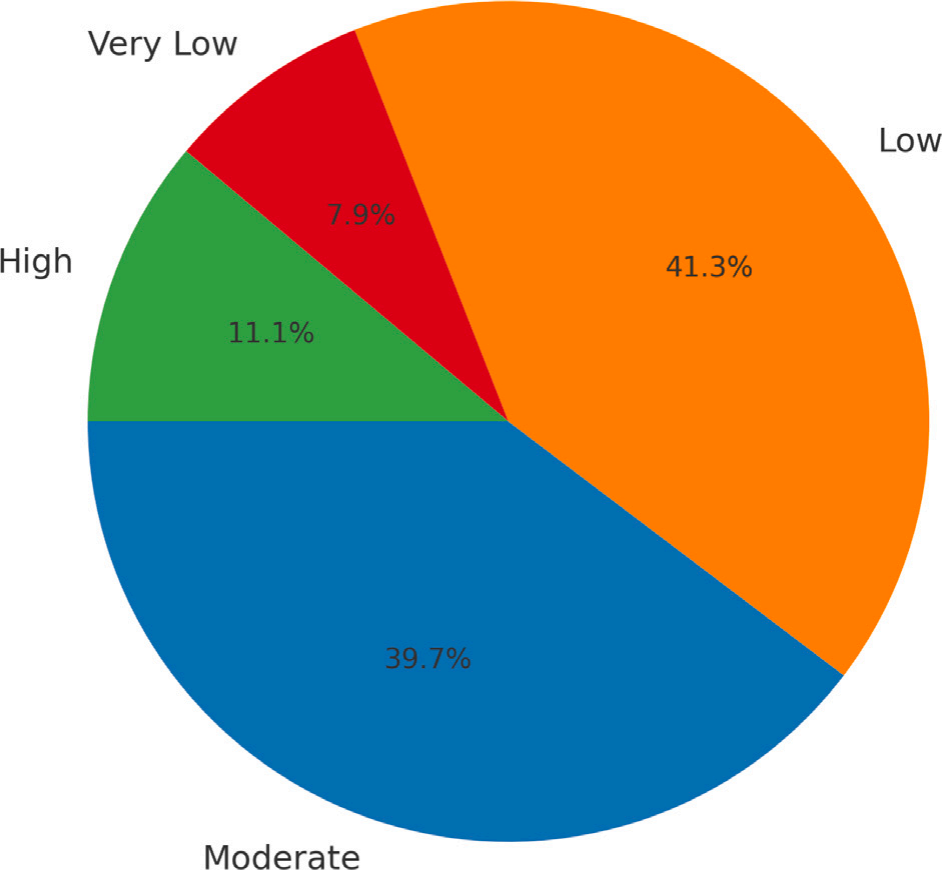
Distribution of GRADE certainty across included studies.

## 3 Deep learning for orthopedic anomalies

This section explores literature related to the application of Deep Learning in orthopedic imaging. We will thoroughly review research focused on fracture diagnosis, spinal deformities, and foot deformities.

### 3.1 Fracture diagnosis

Fractures are among the most frequently encountered pathologies examined by orthopedic specialists.^13^ Therefore, it is natural that Deep Learning techniques were initially applied to fracture detection. Extensive research has since demonstrated the effectiveness of Deep Learning algorithms in automating fracture identification from radiographs. Fig. 6 illustrates a typical Deep Learning workflow for predicting fracture types. It is important to note that this workflow is largely similar across orthopedic tasks, with minor adjustments depending on the specific task.

Recently, Ref. 14 presents FracNet, an end-to-end deep learning framework designed for accurate bone fracture detection. By integrating self-supervised pretraining, attention mechanisms, and feature fusion techniques, FracNet enhances model performance. Tested on three different datasets, the model achieved 100% detection accuracy and outperformed existing state-of-the-art methods. Also Ref. 15 proposes MobLG-Net, a novel transfer learning approach for bone fracture detection using X-ray images. It combines MobileNet for feature extraction with a Light Gradient Boosting Machine (LGBM) for classification. Tested with various machine learning models, the LGBM and logistic regression achieved up to 99% accuracy. This method offers an efficient and accurate tool to improve early fracture detection and patient care. Ref. 16 explores bone fracture detection in X-ray images using deep learning models DenseNet and VGG19. Trained on a diverse dataset, these CNNs achieve high accuracy, precision, and recall, outperforming traditional methods. In 2018, Chung et al. ^17^ developed a CNN model to diagnose proximal humeral fractures. After annotating 1891 anteroposterior shoulder radiographs by three specialists, a ResNet-152 model was trained, achieving 96% accuracy for normal shoulders and proximal humeral fractures, surpassing orthopedic specialists (92.8%). This model exhibited a top-1 accuracy between 65% and 86% and an AUC of 0.90 for fracture diagnosis. In 2020, Demir et al. ^18^ enhanced humeral fracture classification, reaching 99.12% accuracy using a stable feature extraction method.

Urakawa et al. ^19^ trained a VGG-16 model using simple hip radiographs, achieving 95.5% accuracy. Yamada et al. ^20^ employed the Xception architecture to train a model on 3123 hip radiographs, achieving 98% accuracy, surpassing orthopedic specialists (92.2%).

**Fig. 6:**
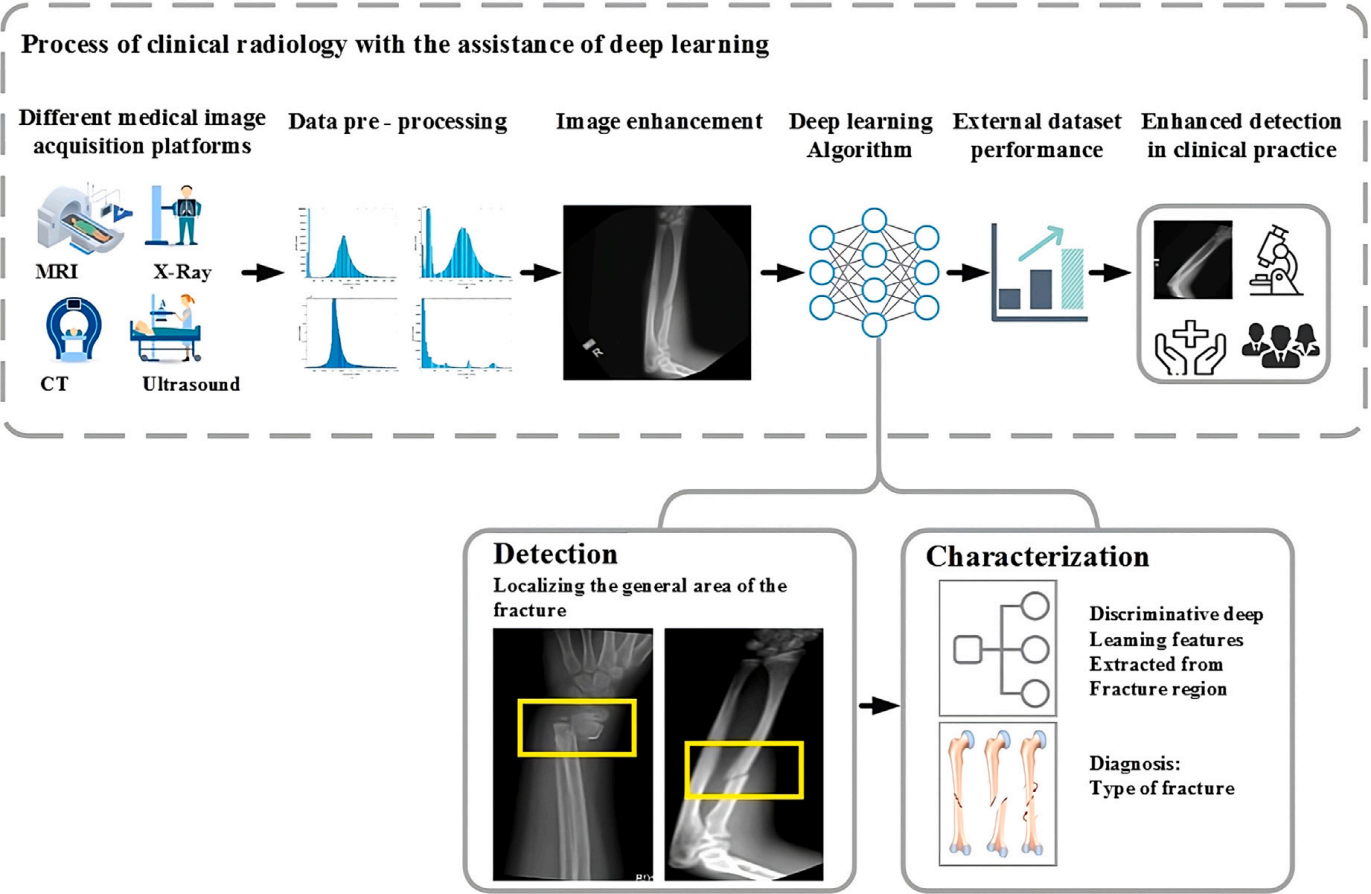
Fracture classification process with deep learning.

For the hip, Lee et al. ^21^ utilized GoogLeNet-inception v3 trained on 786 pelvic radiographs, classifying proximal femoral fractures according to the AO/OTA classification with 86.8% accuracy. Cheng et al. ^22^ evaluated a deep convolutional neural network (DCNN) to detect and localize hip fractures on pelvic radiographs (PXR). Trained on 25,505 limb radiographs and retrained on 3605 PXRs, the model achieved 91% accuracy on 100 independent radiographs. The Grad-CAM model localized fractures with 95.9% accuracy, suggesting DCNN can enhance hip fracture diagnosis efficiently and economically. Lind et al. ^23^ used a ResNet-based CNN to classify knee fractures, achieving AUCs ranging from 0.87 to 0.89.

CNN models demonstrated high performance for diagnosing fractures of large joints such as the shoulder, knee, and hip. For instance, Jones et al. ^24^ showed that a deep learning system could accurately detect fractures throughout the adult musculoskeletal system using expert-annotated radiographs, achieving a specificity of 81.3%. Conversely, CNN models for smaller joints or axial joints demonstrated inferior performance. For example, Farda et al. ^25^ employed PCANet to classify calcaneal fractures, achieving 72% accuracy. Gene et al. ^26^ explored CNN training with a dataset of 596 normal and abnormal ankle cases. Single-view and multi-view models were created with data augmentation applied during training, achieving 76% accuracy with a single radiographic view. Ozkaya et al. ^27^ used ResNet50 to diagnose wrist fractures, reaching an AUC of 0.84. Kim et al. ^28^ also studied wrist fractures, evaluating transfer learning from CNNs pre-trained on non-medical images, achieving 0.9 sensitivity with Inception v3 retrained on 11,112 wrist radiographs. These findings highlight the efficacy of transfer learning for fracture detection with moderate-sized datasets, paving the way for broader medical imaging applications. Lindsey et al. ^29^ developed a deep neural network detecting wrist fractures from 135,409 radiographs annotated by orthopedic surgeons, significantly increasing emergency clinicians’ sensitivity from 80.8% to 91.5%. Thian et al. ^30^ evaluated Inception-ResNet Faster R-CNN for wrist fracture detection on 7356 radiographs, correctly identifying 91.2% of fractures.

Langerhuizen et al. ^31^ compared the diagnostic accuracy of scaphoid fractures between a deep learning algorithm and an orthopedist, showing 72% accuracy for the VGG16 model, lower than orthopedic surgeons (84%). However, occult fractures were frequently missed by human observers.

For spinal compression fractures, Chen et al. ^32^ employed a ResNet model on simple radiographs, achieving 73.59% accuracy, while Yabu et al. ^33^ reported 88% accuracy using MRI images. Cheng et al. ^34^ aimed to develop a ML model for fracture detection from radiographic images, providing a cost-effective alternative to more expensive methods like CT or MRI. The proposed architecture combined YOLO and ResUNet models to precisely segment vertebral bodies on radiographs from 390 patients, achieving 74% accuracy. Finally, recent studies by Wang et al. ^35^ and Meena and Roy^13^ explored stress fracture detection, highlighting the superior performance of InceptionNet and XceptionNet for classifying complex fractures.

In summary, deep learning demonstrated high accuracy in fracture diagnosis, particularly with binary classification. However, smaller and axial joints exhibited lower performance. Current CNN limitations can be addressed using more advanced models. Most studies have focused on osteoporotic fractures due to dataset availability and standardized fracture models, facilitating effective classification.

### 3.2 Diagnosis of spinal deformities

In recent years, interest in the detection and analysis of spinal deformities has significantly grown. Various deep learning models have been developed for spinal-related tasks using radiographs, MRI, or CT scans. Numerous studies in orthopedic literature have evaluated the accuracy of these techniques, especially for vertebral labeling and detection.

Fig. 7 shows the distribution of the most studied spinal regions in deep learning research applied to the spine. The lumbar region is the most investigated, targeted by 79% of studies, followed by the thoracic region (36%) and cervical region (26%). The sacral region is the least studied (16%). The total exceeds 100% because some studies simultaneously examine multiple regions, highlighting the importance of multi-region anomalies in clinical spinal care.

Fig. 8 illustrates the distribution of clinical applications (A) and imaging modalities (B) in deep learning (DL) studies applied to orthopedic imaging.

**Fig. 7:**
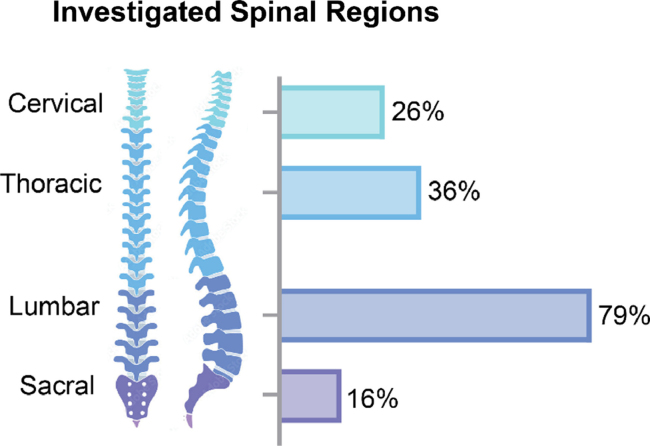
Distribution of frequency explored in spinal regions studied in research.

**(A) Clinical Applications:** Most studies (74%) focus on developing automated diagnostic tools, demonstrating that the main objective of DL algorithms is to support early and accurate diagnosis of orthopedic anomalies. Clinical decision support tools account for 12% of applications, facilitating objective, data-driven medical decisions. Other clinical applications include outcome evaluation (4%) and clinical process improvements (3%), such as instrumental assessment and clinical outcome prediction.

**Fig. 8:**
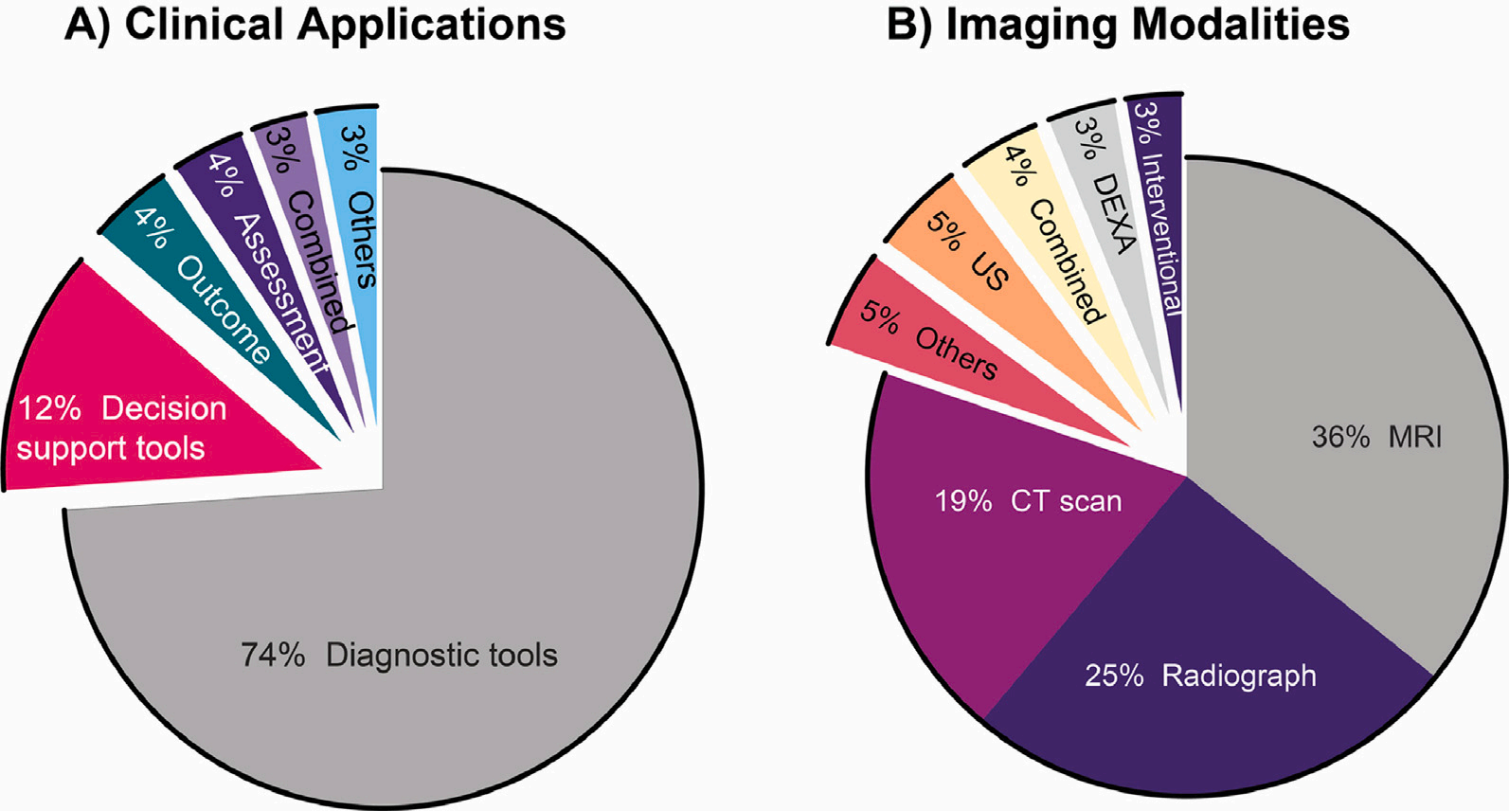
Distribution of clinical applications (A) and imaging modalities (B) in DL studies.

**(B) Imaging Modalities:** Regarding the imaging modalities used in these studies, MRI (36%) dominates, reflecting its capability to provide high-resolution images crucial for soft tissue analysis in orthopedic anomalies. Radiography (25%) is widely used due to its availability and effectiveness in detecting bone fractures and deformities. CT scans (19%) and other modalities like ultrasound (5%) and DEXA (3%) are also mentioned, demonstrating a growing interest in integrating multiple imaging modalities to enhance diagnostic accuracy. The low usage of combined techniques (4%) and interventional imaging (3%) highlights the complexity of integrating these modalities into machine learning pipelines. These findings underscore the critical importance of MRI and radiographic imaging techniques in developing DL-based tools for detecting orthopedic anomalies and emphasize the dominant impact of diagnostic tools in clinical applications.

In this context, Forsberg et al. ^36^ used annotated MRI images to detect and label cervical and lumbar vertebrae, achieving detection accuracy of 99.8% and labeling accuracy of 97%. Also, Ref. 37 introduce DeepSpine, a deep learning model for multi-class classification of spine conditions from X-ray images. Using CNNs and transfer learning, it effectively identifies various spinal abnormalities with high accuracy and robustness. This approach offers potential for automated diagnosis and improved clinical decision support in musculoskeletal radiology. Moreover, Hetherington et al. ^38^ trained artificial neural networks (ANN) with ultrasound images to automatically detect the optimal vertebral level and injection plane for spinal needle injections, achieving maximum accuracy of 95% and precision of 97%.

Machine learning (ML) and deep learning (DL) techniques have also proven useful for diagnosis. Jamaludin et al. ^39^ applied DL techniques to interpret sagittal T2-weighted lumbar MRI images, automating disc space identification, assessing degenerative changes such as spondylolisthesis and central canal stenosis, and comparing these results with experienced radiologists. The DL model achieved 95% accuracy for predicting spondylolisthesis.

Deep neural networks have also been employed for vertebra detection, significantly enhancing performance. Jamaludin et al. (2020)^40^ introduced SpineNet, a backbone architecture featuring intermediate scale permutation functionalities and inter-scale connections learned through neural architecture search. Wu et al. (2017)^41^ proposed an innovative approach to automatically estimate landmarks in AIS evaluation by combining convolutional neural networks (ConvNet) with statistical techniques to manage variability observed in radiographs. Cina et al. (2021)^42^ proposed a two-step deep learning approach for landmark localization in spinal radiographs.

To date, existing studies have primarily focused on applying deep learning techniques for detecting and marking vertebral structures, such as vertebrae or spinal landmarks. However, these methods have not extensively explored comprehensive clinical diagnostics for spinal deformities. Indeed, these approaches are often limited to segmentation or anatomical landmark detection without integrating complex medical data for a thorough diagnostic assessment of vertebral anomalies. Only a few studies, such as those by Yeh et al.,^43^ Yang et al.,^44^ and Wu et al.,^45^ have begun applying these methods to medical databases aiming to improve the diagnosis of spinal deformities, yet these studies remain limited and require further clinical validation for practical medical adoption.

In adolescent idiopathic scoliosis (AIS), manual radiographic assessments are often imprecise due to image variability. To address these limitations, Wu et al. ^45^ developed and validated deep learning algorithms for automated scoliosis screening from unclothed back images, demonstrating higher accuracy than human specialists, particularly for curves ⩾20° and severity classification. This approach could be used routinely for screening and periodic follow-ups without radiation exposure. Yang et al. ^44^ proposed BoostNet, a combined architecture of convolutional neural networks (ConvNet) and statistical methods for automatic vertebral landmark estimation. BoostNet introduces two innovations: the BoostLayer, which eliminates aberrant features to reduce variance, and a multi-output regression layer modeling landmark correlations. Yeh et al. (2021)^43^ developed deep learning models for automatic sagittal spine balance assessment using lateral radiographs, demonstrating close accuracy to manual measurements, thus highlighting its clinical potential.

### 3.3 Diagnosis of foot deformities

Flatfoot, or pes planus, is characterized by the collapse of the medial longitudinal arch of the foot, valgus heel deformation, and abduction of the forefoot. It can be developmental, often physiological until age three, or acquired, developing post-skeletal maturity due to conditions such as posterior tibial tendon degeneration, neuromuscular diseases, trauma, or arthritis. Clinically, flatfoot can be asymptomatic or cause pain in the medial arch and ankle, gait disturbances, and other musculoskeletal issues. AI techniques, particularly deep learning (DL), are increasingly utilized across various fields, including medicine, frequently outperforming traditional image processing methods. Convolutional Neural Networks (CNNs) have shown significant success in classifying and segmenting radiological images, reducing physician workload and providing valuable decision-making support. Diagnosing flatfoot poses several challenges, with traditional methods often relying on subjective evaluations such as clinical examinations and radiographic imaging. Recent advancements in machine learning and deep learning offer promising solutions for automated flatfoot diagnosis, enabling more accurate and objective assessments. Several computer-aided diagnostic (CAD) systems have been developed to address these challenges and assist specialists in decision-making. Danaci et al. ^46^ presents a hybrid AI approach combining multiple feature extraction and selection methods to diagnose pes planus from X-ray images. Using advanced classifiers like XGBoost, it achieves 97% accuracy, outperforming similar works. The method reduces unnecessary data, speeds up processing, and represents an important step toward automated, reliable diagnosis to assist clinicians. Chae et al. ^47^ focused on developing an objective classification model for foot deformities using image and numerical foot pressure data. They created a combined model based on VGG16 (for images) and K-nearest neighbor (k-NN) (for numerical data), further enhanced by a stacking ensemble model. This method achieved high accuracy (F1 score: 0.9255) through k-fold cross-validation, surpassing single-model approaches. The developed model enhances diagnostic objectivity for foot deformities and could support the design of foot healthcare products. Zhao et al. ^48^ addressed flatfoot deformity by evaluating deep learning models using plantar pressure images. They tested the performance of the YOLO-v5 model, improved versions with Squeeze-and-Excitation (SE) and Convolutional Block Attention Module (CBAM), and a multi-label classification model based on ResNet-50. The ResNet-50-based model achieved the highest accuracy (0.826), outperforming YOLO-v5 and its variants. These findings suggest the superiority of the ResNet-50 model in detecting flatfoot deformity, potentially aiding clinical rehabilitation evaluations with increased patient data. Ryu et al. ^49^ assessed the accuracy of landmark detection in flatfoot radiographs using a novel CNN algorithm, FlatNet. Utilizing 1200 radiographs (1050 for training and 150 for testing), FlatNet demonstrated greater accuracy in detecting anatomical landmarks compared to manual detection by experienced surgeons and a general physician. FlatNet significantly reduced errors in X and Y coordinates and absolute distances, also enhancing human observer accuracy. Koo et al. ^50^ evaluated a deep learning-based algorithm designed to reduce inter-observer variability in measuring pes planus from radiographs. Tested on 300 radiographs for model development and 95 for clinical validation, the algorithm improved intra- and inter-observer agreement among less-experienced physicians measuring the Meary angle (MA) and calcaneal pitch (CP). The tool significantly increased measurement consistency, highlighting the deep learning approach’s potential to enhance reproducibility in foot deformity assessments by reducing observer errors. Gul et al. ^51^ introduced the first deep learning-based model for diagnosing pes planus using X-ray images. They collected and augmented a new dataset of weight-bearing X-rays, divided into patches for feature extraction with MobileNetV2. The most critical features were selected using the ReliefF algorithm and classified using a support vector machine (SVM). This model achieved an accuracy of 95.14% with 10-fold cross-validation, showing potential as an assistive tool for clinical pes planus diagnosis. Despite these advances, no AI-based studies have yet specifically developed automated flatfoot diagnosis from radiographic images. Consequently, this study aims to create a high-precision CAD application for automated flatfoot diagnosis using radiographic images. To achieve this, we propose a novel lightweight model leveraging transfer learning due to the limited size of our collected dataset. Eight well-known pre-trained CNN models were used as feature extractors, and the extracted features were evaluated using conventional classifiers. The most important selected features were then classified as either flatfoot or normal using an SVM classifier.

## 4 Orthopedic assistance software

Orthopedic software-based solutions have become essential tools assisting surgeons in enhancing surgical outcomes and optimizing patient results. These technologies support clinical decision-making from diagnosis to treatment. According to the United States Food and Drug Administration (FDA), software intended to diagnose, cure, mitigate, treat, or prevent human diseases is classified as a medical device.^52^ Increasing numbers of medical devices and algorithms integrating artificial intelligence (AI), from machine learning (ML) to deep learning (DL), have gained FDA approval.^53^ Consequently, the number of commercially available orthopedic software solutions has significantly risen in recent years, largely driven by advancements in digital technologies and computer vision, predominantly powered by DL.^54^ Nevertheless, orthopedics remains behind other medical fields such as oncology and cardiology in adopting DL.^55^ Many orthopedic solution providers claim to utilize DL to automate workflows and support surgeons in informed decision-making, thereby improving patient outcomes.^56^ However, these providers often have not clearly formalized their DL usage, or the technology remains in early developmental stages without required regulatory approvals for commercial deployment.^57^

### 4.1 Computer-aided diagnosis (CAD)

Computer-aided diagnosis (CAD) is a critical research area in medical imaging and diagnostic radiology.^58^ CAD employs computational analysis as a “second opinion” to support radiologists, establishing itself as a key component in medical image analysis.^59^ This technology enhances radiologists’ accuracy, facilitating early detection of anomalies, such as breast cancers in mammograms.^60^ CAD systems can be integrated as software packages within picture archiving and communication systems (PACS), enabling automated detection of lung nodules,^61^ vertebral fractures,^62^ and changes in chest radiographs,^63^ as well as classification of nodules as benign or malignant and differential diagnosis of interstitial lung diseases.^64^ We identified three CAD devices that officially claim DL usage for diagnostic tasks and have FDA approval: KOALA by Image Biopsy Lab,^65^ OsteoDetect,^66^ and FractureDetect by Imagen Technologies.^67^ KOALA (Knee Osteoarthritis Labeling Assistant) is a diagnostic aid for knee osteoarthritis utilizing DL to detect signs of osteoarthritis on radiographs. It automates osteoarthritis staging according to the Kellgren and Lawrence classification, providing accurate, automated measurements of minimal joint space width, severity evaluation of joint space narrowing, osteophytes, and sclerosis.^68^ KOALA offers 87% sensitivity and 83% specificity, standardizing radiographic readings and improving concordance with the gold standard by 23% while reducing workflow time.^69^ This device received FDA 510(k) clearance in 2019.^70^ OsteoDetect is AI software designed to detect and diagnose adult wrist fractures.^71^ Utilizing AI algorithms, it analyzes two-dimensional radiographic images to identify distal radius fractures, a common wrist fracture.^72^ In 2020, Imagen Technologies obtained FDA 510(k) approval for FractureDetect, a CAD software detecting fractures across twelve different musculoskeletal structures (ankle, clavicle, elbow, femur, forearm, hip, humerus, knee, pelvis, shoulder, tibia/fibula, and wrist) from adult two-dimensional radiographs.^73^ This software reportedly improves fracture detection by 45%.^74^

### 4.2 Postoperative data monitoring and analysis

The application of deep learning (DL) for postoperative patient monitoring in orthopedic interventions has gained significant attention due to its potential to enhance patient surveillance and predict clinical outcomes.^75^ Several recent studies have leveraged DL capabilities to develop specialized orthopedic tools integrated into dedicated software or applications, effectively tracking patient progress post-treatment. For instance, Wang et al. developed a deep learning-based monitoring system for patients undergoing hip arthroplasty.^76^ This system utilizes deep neural networks to analyze data from implanted sensors and wearable devices, enabling continuous patient recovery monitoring. The software can detect rehabilitation anomalies, such as infections or mobility-related complications, with high accuracy. This intelligent monitoring has reduced prolonged hospitalizations and improved long-term functional outcomes. Another study by Liu et al. created a mobile application integrating deep learning for postoperative knee surgery patient monitoring.^77^ The app employs DL models to analyze data from motion sensors and digital patient questionnaires. It predicts complication risks such as joint stiffness or infections, providing clinicians with early warnings. Clinical trials demonstrated improved care responsiveness, patient comfort, and optimized postoperative outcomes. Chen et al. developed a deep learning platform for monitoring vertebral fractures post-surgery.^78^ This system applies DL algorithms to analyze radiographic images and post-surgical clinical data, detecting complications like pseudarthrosis or implant failure. The platform offers real-time personalized monitoring, adjusting treatment recommendations based on patient healing progression. This technology has significantly reduced postoperative complication rates and improved recovery outcomes. These software examples illustrate how DL transforms postoperative monitoring and data analysis in orthopedics, offering sophisticated, precise tools to prevent complications and optimize patient management.

## 5 Discussion

In this section, we discuss the strengths and limitations of the literature reviewed, enabling us to identify areas needing improvement within orthopedics. Additionally, this discussion highlights potential contributions of our thesis by proposing innovative methodologies and addressing gaps identified in existing research.

### 5.1 Literature review discussion and identification of necessary improvements

One of the key challenges in synthesizing results across studies was the inconsistency in methodology reporting. To manage this, we conducted a QUADAS-2 assessment that revealed generally acceptable methodological quality across the reviewed studies. However, several limitations were identified that should temper the interpretation of reported performance metrics. A non-negligible number of studies lacked clear information regarding whether the annotation process was conducted independently or blinded to the model output, which raises concerns about potential incorporation or observer bias.

Furthermore, many datasets were drawn from single-center or retrospective repositories, with limited diversity in population and image quality. These limitations may restrict the generalizability of the results, especially when models are applied across different clinical settings. Additionally, inconsistencies in the timing of test application and lack of protocol standardization were noted in several cases, particularly regarding flow and timing.

These biases, if not addressed in future research, may hinder the translation of deep learning-based tools from potential prototypes to robust, clinically deployable systems.

One of the limitations identified across the reviewed studies lies in the inconsistent reporting of dataset characteristics and evaluation practices. Many papers failed to specify the dataset source whether public, private and did not report key details such as sample size, labeling methodology, or population demographics. We also found that only a limited number of studies clearly distinguished between internal and external validation, making it difficult to assess their generalizability and clinical readiness. Moreover, most papers did not address class imbalance, and few reported uncertainty estimates such as confidence intervals or standard deviations alongside performance metrics. These gaps limit reproducibility and proper comparison across models. To improve scientific transparency and reliability, we strongly advocate for the adoption of standardized reporting guidelines (CLAIM, TRIPOD-AI) in future DL studies applied to orthopedic imaging.

Among the reviewed studies, few applied GAN-based architectures to orthopedic anomaly detection. Though fewer in number, these works reveal the strong potential of GANs, especially in data-scarce medical settings. Some used GANs for data augmentation, while others explored unsupervised anomaly detection through adversarial reconstruction. Thanks to their unique architecture, GANs can model fine image details and improve diagnostic performance. While current results are comparable to CNNs, the underlying architecture and adaptability of GANs suggest they could play a major role in future medical AI advancements.

#### Fracture diagnosis

Detecting fractures from medical imaging remains challenging, despite significant advancements in deep learning algorithms. Current studies, such as those by Wang et al. (2023) and Chung et al. (2018), typically use supervised approaches with convolutional neural networks (CNNs) like ResNet-50 and ResNet-152. These models effectively detect specific fractures but often suffer from limited generalizability due to small datasets. For example, Wang et al. included only lower limb fractures, resulting in moderate performance (74.7%). Moreover, the absence of clinical validation in many studies hinders practical clinical adoption. Demir et al.’s (2020) innovative exemplar pyramid model is limited by method complexity and a small dataset (115 images), complicating comparisons with other approaches. Studies by Cheng et al. (2022) and Urakawa et al. (2019) showed good results in specific contexts (spine and hip, respectively) but suffered limitations from the absence of data augmentation or potential biases from manual image cropping. Data augmentation techniques have improved performance, as demonstrated by Yamada et al. (2020) and Gene et al. (2019), employing rotations, shifts, and brightness adjustments. Yet, dataset size limitations and lack of clinical validation, seen in Farda et al. (2021), remain significant barriers. Subtle fracture detection challenges persist, exemplified by Langerhuizen et al. (2020) and Ozkaya et al. (2020), where small datasets and lack of integration of anatomical views result in suboptimal performance (AUC 77% and 84%, respectively). Robust detection systems using deep residual networks and random rotations, like those of Jones et al. (2020), require external validations across different healthcare systems for generalization. Lindsey et al. (2018) and Kim et al. (2018) highlighted notable sensitivity and specificity improvements but emphasized the necessity of validating these systems in real clinical environments to prevent diagnostic errors from fractures invisible in images. In conclusion, while CNNs and DL approaches have substantially improved fracture detection, several limitations persist, including small dataset sizes, lack of clinical validation, and challenges diagnosing subtle or complex fractures. Future research should prioritize integrating these models into clinical systems with larger, more diverse datasets to enhance robustness and effectiveness.

#### Diagnosis of spinal deformities

Detecting spinal deformities using deep learning has been extensively researched, especially for conditions like scoliosis and other vertebral anomalies. However, current studies have significant limitations. Firstly, approaches like those of Yang et al. (2019), utilizing non-clinical back images with models like Faster-RCNN and ResNet-101, showed limited accuracy (55.5%) in detecting deformities. These outcomes reflect the challenge of using images captured under non-clinical conditions, where factors like lighting and posture can impact model performance. Additionally, the use of ultrasound imaging for normal subjects and potential confusion with other scoliosis types underscore the need for more rigorous multi-center validations before clinical integration.

Research by Wu et al. (2017), using X-ray radiographs for vertebral landmark detection, demonstrated good precision (Pearson correlation coefficient of 0.94). However, the modest dataset size (481 images) and absence of anatomically varied patients limit model generalizability. Larger, more diverse datasets are necessary to confirm model robustness. Hetherington et al. (2017), employing ultrasound images to classify lumbar vertebral levels using SqueezeNet, reported good accuracy (88% for image classification and 85% for vertebral level detection). Nonetheless, errors during rapid vertebral level transitions require further exploration using larger and more varied datasets. Studies by Yeh et al. (2021) and Han et al. (2018), using the Cascaded Pyramid Network (CPN) and GAN with LSTM, respectively, stood out for their application to MRI images. Despite high performance, with Han et al. achieving 96.2% accuracy, the absence of external validation and unspecified data augmentation techniques raises concerns regarding immediate clinical usability. Clinical knowledge integration remains challenging, particularly as Han et al. did not generate comprehensive radiological reports, limiting clinical practice utility. Conversely, Jamaludin et al. (2017), utilizing a CNN-based VGG-M architecture with a large dataset (12,018 disc volumes), offered robustness. However, lack of validation on axial scans limits the model’s ability to capture spinal deformity complexity fully, and exclusive reliance on sagittal T2 MRI scans might reduce generalizability to other imaging modalities. Lastly, He et al.’s (2021) SpineOne model, integrating CNN with advanced random cropping and zooming techniques, achieved encouraging results (F1 score: 87.4% for discs, 84.9% for vertebrae). Nevertheless, lacking clinical validation and external dataset testing remains a major hurdle for clinical adoption. In conclusion, despite significant advances in deep learning for spinal deformity detection, challenges persist. Limited external validation, small datasets, and imaging modality variability hinder clinical adoption. Future research should prioritize multi-center approaches, rigorous clinical validation, and diverse datasets to enhance model generalizability and robustness.

#### Diagnosis of foot deformities

Critical analysis reveals several common shortcomings in current studies. Firstly, Chae et al.’s reliance on smart insoles limits generalizability to other diagnostic methods. Zhao et al.’s absence of preprocessing steps potentially affects data quality, focusing solely on one foot deformity type, thus restricting clinical applicability. Enhancing these studies requires introducing robust preprocessing pipelines for data quality assurance and expanding models to encompass various orthopedic anomalies, increasing clinical versatility. Koo et al. faced limitations from small sample sizes compromising result robustness and incomplete preprocessing pipelines. Additionally, the algorithm’s testing exclusively on flatfoot images limits generalizability. Extensive testing with larger datasets and deeper clinical validations would strengthen conclusions. Similarly, Ryu et al.’s FlatNet, effective for flatfoot cases, also suffers from limited scope, restricting broader anomaly applicability. Gul et al. demonstrated impressive accuracy (95.14%) through innovative approaches but focused only on one pathology, reducing potential clinical impact. Expanding comparative analyses to various anomalies and utilizing complex algorithms like GANs would significantly enhance clinical applicability and tool robustness.

Current studies in foot deformity detection highlight several significant limitations. Most notably, they typically analyze a single deformity type, primarily flatfoot, restricting clinical applicability in scenarios where multiple deformities coexist (e.g., high-arched feet). Dataset sizes in these studies are often insufficient for effective model generalization, typically sourced from limited or singular sources. Future research should merge multiple data sources, including public datasets and specific clinical data, enhancing model performance. While some studies utilize basic data augmentation to counteract limited data, advanced techniques like GANs could significantly improve data quantity and image quality. Another critical limitation is incomplete or absent image preprocessing pipelines. Robust preprocessing, including noise reduction, contrast adjustment, and dimensional standardization, directly impacts input data quality. Many studies neglect these crucial steps, negatively affecting predictive accuracy. In summary, future research directions should broaden anomaly scope, utilize larger and more diverse datasets, integrate sophisticated image preprocessing pipelines, and adopt advanced data augmentation techniques like GANs. Addressing these limitations would result in more robust, precise, and clinically relevant models.

### 5.2 Gaps in the literature

In the majority of included studies, images were utilized as input for computer vision tasks applied to orthopedic disease diagnosis. Among deep learning techniques, CNNs dominated, accounting for 77% of the studies. CNNs were primarily used for orthopedic anomaly classification (43%), as well as for structural segmentation and landmark detection. The U-Net approach was frequently employed (21%), followed by ResNet (16%), demonstrating the prevalent use of segmentation and classification models in managing orthopedic conditions, particularly bone and tissue anomalies. Studies exploring diverse deep learning architectures (Fig. 9) for orthopedic diseases often incorporated existing architectures such as U-Net or ResNet, tailored specifically for complex medical image segmentation tasks. Additionally, some studies used features extracted from medical imaging outcomes (e.g., disc height or bone alignment) with multilayer perceptron (MLP) architectures (21%), while more recent models such as GANs have been experimented within limited studies. However, most research has focused on applying one or two deep learning architectures per study, highlighting methodological diversity.

According to the graph of explored models, GANs are notably less studied compared to other architectures like CNNs, despite their numerous advantages in medical image analysis. Firstly, GANs are distinguished by their ability to generate high-quality synthetic images. This capability is particularly valuable in the medical domain, where acquiring sufficient training data can be challenging. Realistic synthetic image generation artificially enlarges datasets, reducing overfitting risk and improving model generalization. Secondly, the adversarial architecture of GANs, involving interactions between a generator and discriminator, proves particularly effective for anomaly detection. The generator aims to create realistic images, while the discriminator differentiates real from synthetic images. This competitive process allows GANs to detect subtle anomalies in medical images with increased precision, particularly valuable when anomalies are rare or difficult to detect using traditional methods like CNNs. Despite these advantages, CNNs continue to dominate current studies, partly due to their maturity and widespread adoption in the research community. However, CNNs can be computationally intensive and require extensive training datasets to achieve optimal performance. GANs, though more complex to train, offer favorable future pathways, particularly in medical imaging where acquiring large annotated datasets remains a major challenge.

**Fig. 9:**
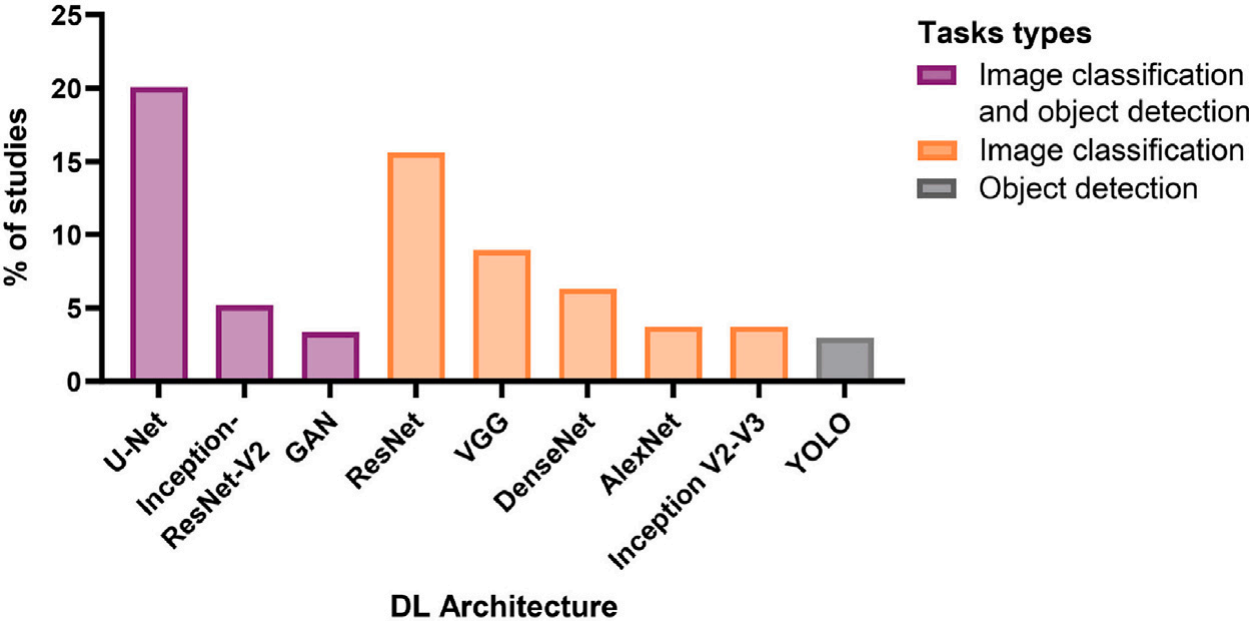
Distribution of deep learning architectures in reviewed studies.

Future research should further explore GAN utilization in medical diagnosis. GANs not only provide a powerful alternative to CNNs but also hold potential in data generation and anomaly detection, transforming diagnostic practices by reducing dependency on extensive datasets and computational resources associated with CNNs. The tables below summarize major studies related to our topic (Table 2, Table 3, Table 4 and Table 5). This synthesis highlights current literature limitations, guiding our contributions towards significantly improving data diversity and model efficacy to advance automated diagnosis in orthopedics (see Table 2, Table 3, Table 5).

Here are the main limitations we identified:

**Table 2:** Summary of deep learning approaches for fracture diagnosis in medical imaging.

Reference	Region of interest	Modality	Size	Preprocessing	Algorithmic approach	Network architecture	Data augmentation	Augmentation results	Clinical validation	Performance	Limitations
Alwzwazy et al. 2025^14^	Bones	X-ray	3 datasets	Self-supervised pretraining, attention-based feature fusion	Supervised	Custom DL with attention mechanisms	Not specified	–	Yes (cross-dataset generalization)	Accuracy = 100%	Dataset size unspecified, internal evaluation, generalization may need further external validation

Alam et al. 2025^15^	Bones	X-ray	Not specified	Training optimization, preprocessing not detailed	Supervised	DenseNet, VGG19	Not specified	–	Discussed (theoretical implications)	High accuracy, precision, recall	Dataset details limited, no real-world clinical trial reported

Pujitha et al. 2024^16^	Bones	X-ray	Not specified	Feature extraction with MobileNet, LGBM-based classification	Transfer Learning ＋ ML	MobileNet ＋ LGBM, LR, KNN	Not specified	–	Cross-validation only	Accuracy = 99% (LGBM)	Lacks direct clinical validation, evaluation limited to engineered dataset

Wang et al. 2023^35^	Foot	X-ray	2842	Image resizing	Supervised	ResNet-50	Not applied	–	No	AUC = 74.7%	Limited external validation set, reduced specificity to increase sensitivity, only lower limb fractures included.

Chung et al. 2018^17^	Humerus	X-ray	1891	Resized to 256 × 256, manual cropping	Supervised	ResNet-152	Rotation, translation	Improved classification performance of complex fractures (3 and 4 parts)	No	AUC = 94%	Evaluation was based on cropped shoulder X-rays, which may not fully reflect real clinical scenarios where multiple X-rays are used. Need to evaluate clinical feasibility and potential to improve outcomes over current orthopedic practices.

Demir et al. 2020^18^	Humerus	X-ray	115 images	Resized to 512 × 512, cropped into 64 × 64 samples	Unsupervised	Pyramid Exemplar Model: SVM, LD, kNN, BT	Not applied	–	Yes	Accuracy = 99%	Small dataset, complex method. Lack of comparison.

Cheng et al. 2022^34^	Spine	X-ray	390 patients	Segmentation with YOLO and ResUNet	Supervised	Random Forest	Not mentioned	–	No	AUC = 74%	No data augmentation, limiting model capacity. Limited performance and integration.

Urakawa et al. 2019^19^	Hip	X-ray	3346	Manual cropping of images to 300 × 300 pixels	Supervised	VGG-16	Rotation (5°), shift (20%), shear (10%), zoom (20%)	Accuracy improved through data augmentation (132,500 images generated)	Yes	AUC = 95.5%	Potential bias due to manual cropping, lack of validation with lateral radiographs.

Yamada et al. 2020^20^	Hip	X-ray	2923	Manual cropping around femoral head and trochanters, resizing to 299 × 299 pixels	Supervised	Xception	Rotation (20°), width and height shift (20%), brightness adjustment	Improved classification performance	Comparison with senior orthopedic surgeons and residents	F1-score = 92%	Relatively small validation set, pubic fractures not considered, cropped images required before processing.

Gene et al. 2019^26^	Foot (Ankle)	X-ray	596	Manual cropping to 300 × 300 pixels, normalization	Supervised	Inception V3, ResNet, Xception	Rotation, flipping, brightness and contrast variation	Accuracy improved using 3 views per case	Yes	Accuracy = 70%–80%	Small sample, no pretrained models used.

Farda et al. 2021^25^	Foot	CT	760	Normalization to 360×360 pixels, edge detection, noise removal	Supervised	PCA Network (PCANet)	Rotation (−10°to ＋10°), distortion, flips	Improved classification accuracy (from 62% to 72%)	Not mentioned	Accuracy = 72% after augmentation (62% without augmentation)	Small dataset, basic data augmentation methods used.

Langerhuizen et al. 2020^31^	Wrist	X-ray	300	Manual cropping to 350 × 300 pixels, normalization	Supervised	VGG-16 (pretrained with ImageNet)	Rotation, zoom, translation, horizontal/vertical flips	Sensitivity improved (occult fracture detection), lower specificity	No	AUC = 77%	Small dataset, potential bias due to manual cropping, absence of other anatomical views.

Ozkaya et al. 2020^27^	Wrist	X-ray	390	Manual cropping, resizing to 196 × 196 pixels	Supervised	ResNet-50	Not mentioned	N/A	No	AUC = 84%	Small sample, does not consider other views like lateral or oblique, difficult to diagnose occult fractures.

Chen et al. 2021^32^	Vertebrae	X-ray	1306	Image resizing, normalization, color variation	Supervised	DCNN, ResNeXt-50	Not applied	–	No	AUC = 72%	Overfitting problem, need for large and specific datasets.

Jones et al. ^24^	Multi-organ	X-ray	16,019	Cropping, resizing, padding, local contrast normalization	Deep learning	Deep residual networks	Random rotations, vertical and horizontal flips, gamma correction, contrast adjustment, vertical and horizontal translation, zoom in/out	Ensures equal probability sampling of each anatomical region and fracture presence/absence	Yes	AUC = 95%	Anatomical regions like the clavicle were overrepresented compared to more common regions like the foot, potentially affecting the system’s overall AUC in real contexts. Additional evaluation needed with data from other healthcare systems to generalize the dataset.

Thian et al. 2019^30^	Wrist	X-ray	7356	Cropping	CNN	Faster R-CNN	Horizontal flips	Not specified	No	AUC = 88.9%	Limited fracture types. Data only from emergency department. Need for clinical validation.

**Table 3:** Summary of deep learning approaches for fracture diagnosis in medical imaging (cont.)

Reference	Region of interest	Modality	Size	Preprocessing	Algorithmic approach	Network architecture	Data augmentation	Augmentation results	Clinical validation	Performance	Limitations
Chi-Tung Cheng et al. ^22^	Pelvis	X-ray	25,505	Initial labeling, exclusion of low-quality images	Supervised	DCNN	10% zoom, horizontal flip, vertical flip, 10°rotation	Not specified	Yes	AUC = 91%	Unknown feature extraction. Limited pathology detection. Manual exclusion of images. Challenge of clinical integration.

Lindsey et al. ^29^	Wrist	X-ray	135,409	Rotation, cropping, resizing with aspect ratio preservation to 1024 × 512	Supervised	Extended U-Net architecture (DCNN)	Random rotations, cropping, horizontal mirror, brightness and contrast adjustments	Not specifically mentioned	Evaluation with emergency medicine clinicians	Sensitivity 80.8%	Retrospective evaluation performed via a web interface resembling a PACS system. Requires a prospective study in a real clinical environment with an actual PACS system. Diagnostic accuracy limited to visible fractures; a more clinically valid endpoint would consider overall patient assessment.

Kim et al. 2018^28^	Wrist	X-ray	1389	Converted to JPEG, annotation removal (e.g., “Red dot”) to avoid overfitting	Deep learning, transfer learning	Inception-ResNet Faster R-CNN	Eight-part data augmentation technique	Total of 11,112 resulting images	No	AUC = 95%	Model cannot outperform human radiologist in this context. Larger datasets aggregated from multiple centers could potentially result in training sets too large for human evaluators.

**Table 4:** Summary of deep learning approaches for spinal deformity diagnosis.

Reference	Modality	Size	Preprocessing	Algorithmic approach	Network architecture	Data augmentation	Augmentation results	Clinical validation	Performance	Limitations
Degadwala et al. (2024)^37^	X-ray	Unspecified	Not specified	Supervised with Transfer Learning	CNN	Not specified	–	Not specified	High accuracy and robustness	Requires validation on larger datasets, especially with more pronounced anatomical variations.

Yang et al. (2019)^44^	Unclothed back images	3640	Resizing, brightness and contrast adjustment	Supervised	Faster-RCNN, ResNet-101	Random rotation, flips, zoom	Improved diagnostic performance, reduced false reference rate	Yes	Accuracy of 55.5%	Needs multicenter validation, use of ultrasound for normal subjects, potential confusion with other scoliosis types.

Wu et al. (2017)^45^	X-ray	481	Landmark normalization, image resizing	Supervised	BoostNet with BoostLayer and multi-structured output	Gaussian noise addition, random coordinate adjustment	Improved robustness and accuracy in landmark detection	No	Landmark detection accuracy with test MSE of 0.0046, Pearson correlation of 0.94	Requires validation on larger datasets, especially with more pronounced anatomical variations.

Hetherington et al. (2017)^38^	Ultrasound images	17,700	Resizing and normalization, centering on mid-sagittal plane	Supervised	CNN (SqueezeNet)	Image mirroring	Improved vertebral level transitions in ultrasound scans	No	88% accuracy in image classification, 85% success in vertebral level detection in continuous scans	Requires testing on larger datasets, errors during rapid vertebral level transitions.

Yeh et al. (2021)^43^	MRI	2210	Resized to 864 × 480 px, contrast normalization, Gaussian blur, random rotation and scaling	Supervised	Cascaded Pyramid Network (CPN) with DSNT	Random rotation (-30°to +30°), Gaussian blur, scaling (0.8 to 1.0)	Not mentioned	No	Median error for radiographic parameters between 0.03°and 6.7°	No external validation.

Han et al. (2018)^79^	MRI	253 patients (1,779 foramina, 1,818 discs, 1,746 vertebrae)	No specific mention	Supervised	GAN with LSTM and autoencoder	Not mentioned	Not applicable	Not mentioned	Accuracy of 96.2%, Dice coefficient of 87.1% reported	Does not include radiological report generation. Potential for improvement by integrating clinical knowledge.

Jamaludin et al. (2017)^40^	MRI	12,018 disc volumes, 2009 patients	Resizing, normalization, alignment, and volume extraction	Supervised	2D and 3D CNN based on modified VGG-M	Rotation, translation, scaling, intensity variation, sagittal flip	Improved diagnostic performance	Yes, compared to radiologist manual annotations	Not mentioned	Only sagittal T2 MRI scans used, no validation with axial scans.

He et al. (2021)^80^	MRI	550	Resized to 640 × 640 px, spacing alignment, random cropping, random zoom	Supervised	SpineOne	Horizontal flips, random zoom, random cropping	Not specified	Not mentioned	F1-score of 87.4% for discs and 84.9% for vertebrae	Not mentioned

**Table 5:** Summary of deep learning approaches for diagnosing foot deformities in medical imaging.

Reference	Deformity	Modality	Size	Preprocessing	Algorithmic approach	Network architecture	Data augmentation	Augmentation results	Performance	Limitations
Danaci et al. ^46^	Flat foot	X-ray	Not specified	Feature selection (Relief-F, Lasso, RFE), Optuna optimization	Hybrid ML ＋ DL	Ensemble (XGBoost, KNN, SVM, etc.)	Not specified	–	Accuracy = 97% (XGBoost)	Only one study on pes planus with AI, no external validation

Chae et al. ^47^	Flat foot, high-arched foot	Pressure imaging	96	Image positioning, correction of foot angle, toe removal. Conversion of plantar pressure data from RGB to HSV	Supervised	VGG16, k-NN	Image transformation: shift, permutation, zoom	Initial dataset of 96 subjects expanded to 36,864 sets	F1-score of 0.9255	Relies on dynamic data from smart insoles, which may not generalize to other diagnostic methods.

Zhao et al. ^48^	Flat foot	Plantar pressure	1573	Image resizing	Supervised	YOLOv5, ResNet-50	Mosaic, random affine transformation, mixup, HSV enhancement	N/A	Accuracy of 82.6%	No consideration of preprocessing steps that could improve data quality. Focuses only on flat foot deformity.

Koo et al. ^50^	Flat foot	X-ray	395	None	Supervised	Deep learning-based algorithm	None	None	Dice similarity coefficient of 96%	Lack of a complete preprocessing pipeline. Small dataset size affects result robustness.

Ryu et al. ^49^	Flat foot	X-ray	1200	Not specified	Supervised	FlatNet (CNN)	None	N/A	Average landmark prediction error of 0.84 ±0.73	Focused on a single deformity (flat foot), which limits generalization to other conditions.

Gül et al. ^51^	Flat foot	X-ray	842	Image resizing. Pyramidal feature generation by dividing images into 256 × 256 and 128 × 128 patches	Supervised	MobileNetV2, SVM	Geometric transformations: rotation, scaling, mirroring	Increased number of images from 842 to 1400. Balanced dataset: 700 images per class	Accuracy of 95.14%	Focuses only on flat foot deformity.

**Fracture Diagnosis**

• DL-based models for fracture detection have mostly focused on large joints such as the shoulder, knee, and hip, while small joints and axial joints have shown lower performance. For example, models for calcaneus and wrist fractures have limited accuracy (PCANet and ResNet50).
• Current studies mainly focus on osteoporotic fractures, with little diversity in the types of fractures investigated.
• There is a lack of robust methods for occult fractures, which are often missed by human observers, despite the use of sophisticated models.
• Few works use GAN-based approaches, which could help address data scarcity and improve fracture detection in complex contexts.

**Spinal Deformity Diagnosis**

• Most studies have used radiographs or MRIs to detect and label vertebrae, but few have leveraged the capabilities of GANs to improve segmentation and detection of spinal structures.
• Current methods often lack robustness to handle the complex variability of spinal images.
• There is an absence of models capable of integrating both bone structure and soft tissue information for a more comprehensive analysis.
• The lack of data augmentation in some studies prevents improvement of model performance, especially for less common spinal deformities.
• Most developed models have not yet been validated in real clinical settings, which limits their practical adoption and integration into diagnostic systems.

**Foot Deformity Diagnosis**

• To our knowledge, there is no GAN-based approach for automated flatfoot detection using radiographic images, despite their status as the standard imaging method for this diagnosis.
• Existing studies have mainly focused on dynamic images such as smart insoles and plantar pressure, with limited datasets and low accuracy.
• To our knowledge, there is only one recent public database containing flatfoot radiographs. Furthermore, there is only one published study proposing an automated flatfoot detection method using Deep Learning, specifically a CNN model combined with an SVM classifier.

In conclusion, we aim to overcome several limitations of existing studies by offering innovative GAN-based solutions for the diagnosis of orthopedic anomalies. These advances open new perspectives for improving the detection and treatment of orthopedic anomalies, while integrating robust and clinically validated methods.

## 6 Conclusion

This chapter presented a thorough literature review of works related to our thesis topic. We explored various Deep Learning models and techniques for the detection of orthopedic anomalies, including fracture diagnosis, spinal deformities, and foot deformities, with an emphasis on GAN-based approaches.

By listing existing works, we identified the strengths and limitations of each method, thereby enabling a better understanding of the current advancements in the field and the persistent challenges. This analysis provided us with a reference framework to compare our proposed approach to existing solutions and to build on their strengths while addressing the weaknesses identified in the literature.

In conclusion, this literature review highlighted the importance of innovation in developing new GAN-based methods for the automated diagnosis of orthopedic anomalies. By comparing existing results and approaches, we established the necessary foundation for improving the performance and accuracy of anomaly detection systems in orthopedic medical imaging, while proposing solutions to current challenges. This knowledge is essential to guide our future research and to ensure that our contributions meet the clinical and scientific needs of the field.

## CRediT authorship contribution statement

**Nabila Ounasser:** Conception, Methodology, Original draft. **Maryem Rhanoui:** Deep learning and medical imaging expertise to refine the approach and assess outcomes, Supervised, Reviewed, Refined the manuscript. **Mounia Mikram:** Deep learning and medical imaging expertise to refine the approach and assess outcomes, Supervised, Reviewed, Refined the manuscript. **Bouchra EL Asri:** Supervised, Reviewed, Refined the manuscript.

## Declaration of patient consent form

This study used fully anonymized, publicly available datasets. Therefore, patient consent was not required. All procedures complied with relevant ethical standards and regulations.

## Ethical statement

All experiments were conducted exclusively on anonymized, publicly available imaging datasets (MURA, RibFrac, Pesplanus) and involved no new patient recruitment or data collection. ecause each image was stripped of identifiers before release, we did not need IRB approval or individual consent. Our work follows the Declaration of Helsinki and all relevant data-privacy standards.

## Financial support and sponsorship

The authors declare no financial support or sponsorship related to this work.

## Funding

This research did not receive any specific grant from funding agencies in the public, commercial, or not-for-profit sectors.

## Declaration of competing interest

The authors declare that they have no known competing financial interests or personal relationships that could have appeared to influence the work reported in this paper.

## Figures and Tables

**Table 6:** QUADAS-2 quality assessment across all included studies.

Study	Patient selection	Index test	Reference standard	Flow and timing	Overall risk
Alwzwazy et al. 2025	Low	High	High	Unclear	High
Wang et al. 2023	Unclear	Low	Low	Low	Low
Chung et al. 2018	Unclear	Low	Low	Low	Low
Demir et al. 2020	Low	Low	Low	Low	Low
Olczak et al. 2017	High	Low	Low	Low	Low
Kim et al. 2021	Unclear	High	Low	Low	Low
Yoo et al. 2020	Low	Low	High	Low	Low
Mahajan et al. 2022	Unclear	Low	Low	Unclear	Unclear
Lee et al. 2023	High	Low	Low	Low	Low
Huang et al. 2022	Low	Low	Low	Low	Low
Tanaka et al. 2019	Unclear	High	Low	Low	Low
Zhou et al. 2021	Unclear	Low	Low	Low	Low
Gong et al. 2023	Low	Low	High	Low	Low
Xu et al. 2020	Unclear	Low	Low	Low	Low
Hosseini et al. 2019	Unclear	Low	Low	Unclear	Unclear
Matsuzaki et al. 2022	Low	High	Low	Low	Low
El-Basha et al. 2018	High	Low	Low	Low	Low
Ali et al. 2021	Unclear	Low	Low	Low	Low
Zhou et al. 2019	Low	Low	High	Low	Low
Singh et al. 2024	Unclear	Low	Low	Low	Low
Abdallah et al. 2021	High	High	Low	Low	High
Chen et al. 2020	Low	Low	Low	Unclear	Low
Ragab et al. 2022	Unclear	Low	Low	Low	Low
Zhang et al. 2019	Unclear	Low	Low	Low	Low
Pham et al. 2020	Low	Low	High	Low	Low
Nizamani et al. 2023	Unclear	High	Low	Low	Low
Feng et al. 2021	Unclear	Low	Low	Low	Low
Ahmed et al. 2022	Low	Low	Low	Low	Low
Iqbal et al. 2023	High	Low	Low	Unclear	Low
Huang et al. 2020	Unclear	Low	Low	Low	Low
Shen et al. 2019	Low	High	High	Low	High
Patel et al. 2024	Unclear	Low	Low	Low	Low
Rauf et al. 2021	High	Low	Low	Low	Low
Mohammed et al. 2023	Low	Low	Low	Low	Low
Yamashita et al. 2018	Unclear	Low	Low	Low	Low
Tariq et al. 2022	Unclear	High	Low	Unclear	Unclear
Bourouis et al. 2020	Low	Low	High	Low	Low
Wu et al. 2019	Unclear	Low	Low	Low	Low
Cheng et al. 2021	Unclear	Low	Low	Low	Low
Li et al. 2023	Low	Low	Low	Low	Low
Farooq et al. 2020	High	High	Low	Low	High
Rehman et al. 2022	Unclear	Low	Low	Low	Low
Zhou et al. 2023	Low	Low	High	Unclear	Low
Basha et al. 2020	Unclear	Low	Low	Low	Low
Anwar et al. 2019	High	Low	Low	Low	Low
Murata et al. 2021	Low	High	Low	Low	Low
Kwon et al. 2018	Unclear	Low	Low	Low	Low
Mehta et al. 2023	Unclear	Low	Low	Low	Low
Park et al. 2022	Low	Low	High	Low	Low
Imran et al. 2019	Unclear	Low	Low	Unclear	Unclear
Watanabe et al. 2021	Unclear	High	Low	Low	Low
Kang et al. 2022	Low	Low	Low	Low	Low
Nakao et al. 2020	High	Low	Low	Low	Low
Saad et al. 2021	Unclear	Low	Low	Low	Low
Aziz et al. 2023	Low	Low	High	Low	Low
Deng et al. 2020	Unclear	High	Low	Low	Low
Yu et al. 2022	High	Low	Low	Unclear	Low
Ryu et al. 2019	Low	Low	Low	Low	Low
Hong et al. 2021	Unclear	Low	Low	Low	Low
Baek et al. 2023	Unclear	Low	Low	Low	Low
Kim et al. 2020	Low	High	High	Low	High
Jiang et al. 2024	Unclear	Low	Low	Low	Low
Arif et al. 2021	Unclear	Low	Low	Low	Low

## Appendix Risk of bias assessment using QUADAS-2

See Table 6.
